# Machine Learning Approaches to Investigate the Structure–Activity Relationship of Angiotensin-Converting Enzyme Inhibitors

**DOI:** 10.1021/acsomega.3c03225

**Published:** 2023-11-08

**Authors:** Tianshi Yu, Chanin Nantasenamat, Nuttapat Anuwongcharoen, Theeraphon Piacham

**Affiliations:** † Center of Data Mining and Biomedical informatics, Faculty of Medical Technology, Mahidol University, Bangkok 10700, Thailand; ‡ Streamlit Open Source, Snowflake Inc., San Mateo, California 94402, United States; § Department of Clinical Microbiology and Applied Technology, Faculty of Medical Technology, Mahidol University, Bangkok 10700, Thailand

## Abstract

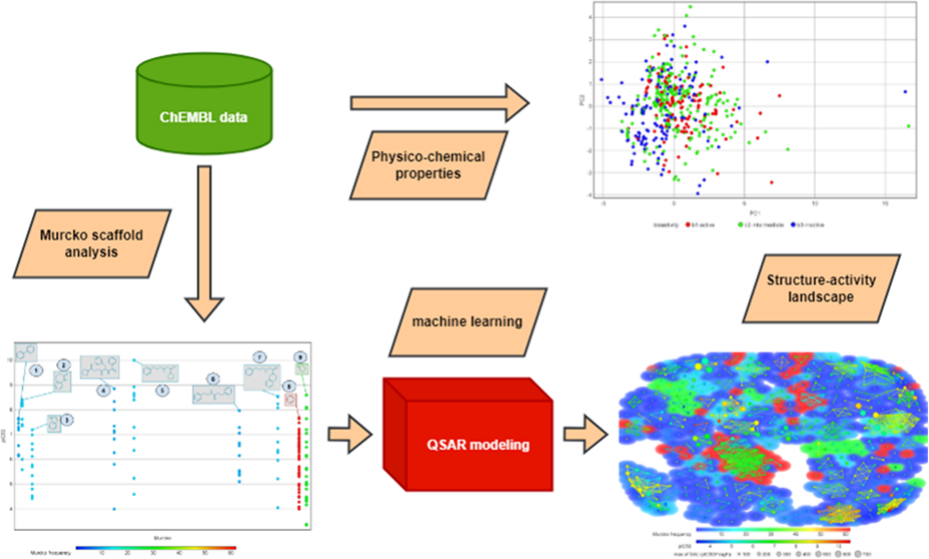

Angiotensin-converting enzyme inhibitors (ACEIs) play a crucial role in treating conditions such as hypertension, heart failure, and kidney diseases. Nevertheless, the ACEIs currently available on the market are linked to a variety of adverse effects including renal insufficiency, which restricts their usage. There is thus an urgent need to optimize the currently available ACEIs. This study represents a structure–activity relationship investigation of ACEIs, employing machine learning to analyze data sets sourced from the ChEMBL database. Exploratory data analysis was performed to visualize the physicochemical properties of compounds by investigating the distributions, patterns, and statistical significance among the different bioactivity groups. Further scaffold analysis has identified 9 representative Murcko scaffolds with frequencies ≥10. Scaffold diversity has revealed that active ACEIs had more scaffold diversity than their intermediate and inactive counterparts, thereby indicating the significance of performing lead optimization on scaffolds of active ACEIs. Scaffolds 1, 3, 6, and 8 are unfavorable in comparison with scaffolds 2, 3, 5, 7, and 9. QSAR investigation of compiled data sets consisting of 549 compounds led to the selection of Mordred descriptor and Random Forest algorithm as the best model, which afforded robust model performance (accuracy: 0.981, 0.77, and 0.745; MCC: 0.972, 0.658, and 0.617 for the training set, 10-fold cross-validation set, and testing set, respectively). To enhance the model’s robustness and predictability, we reduced the chemical diversity of the input compounds by using the 9 most prevalent Murcko scaffold-matched compounds (comprising a total of 168) followed by a subsequent QSAR model investigation using Mordred descriptor and extremely gradient boost algorithm (accuracy: 0.973, 0.849, and 0.823; MCC: 0.959, 0.786, and 0.742 for the training set, 10-fold cross-validation set, and testing set, respectively). Further illustration of the structure–activity relationship using SALI plots has enabled the identification of clusters of compounds that create activity cliffs. These findings, as presented in this study, contribute to the advancement of drug discovery and the optimization of ACEIs.

## 1 Introduction

Angiotensin-converting enzyme (ACE) is an important enzyme of the renin–angiotensin–aldosterone system (RAAS). ACE is responsible for converting inactive angiotensin I into active angiotensin II, a process that results in vasoconstriction and, consequently, an increase in blood pressure. ACE also catalyzes the degradation of bradykinin, a function that contributes to vasodilation, natriuresis (the removal of sodium ions through urine), and regulation of oxidative stress. There are two types of ACE enzymes distinguished by their locations: somatic ACE and germinal ACE. Somatic ACE enzymes are primarily found in the capillaries of the lungs as well as in endothelial and epithelial cells of the kidney, whereas germinal ACE enzymes are predominantly present in sperm cells. Somatic ACEs make up the majority of ACEs and are characterized by having two homologous catalytic domains, namely, the C-domain and the N-domain. The C-domain primarily plays a role in blood pressure regulation, while the N-domain is involved in the control of hematopoietic stem cell proliferation.^1^ The currently available ACE inhibitors (ACEIs) are associated with certain adverse effects, which can be attributed to their nonselective inhibition of both domains. Furthermore, specific inhibitors targeting the N-domain have emerged as potential candidates for antifibrotic drugs.^2^

**Figure 1:**
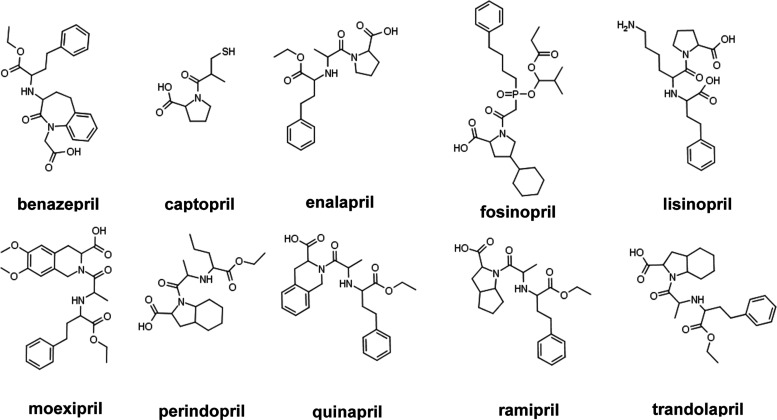
List of FDA-approved ACEIs.

ACEIs are primarily prescribed for the management of hypertension and heart failure. However, they are also commonly utilized in the treatment of kidney diseases.^3^ The majority of ACEIs currently available are short peptides and their chemical derivatives. Captopril, derived from snake venom, was the first ACEI to be discovered, and since its introduction, there have been a series of peptide derivatives developed as ACEIs. These peptide-derived ACEIs typically share a common core structure that includes a group capable of chelating the divalent zinc ion at the ACE active site. In addition, there are more than 10 ACEIs that have been marketed as shown in Figure 1. Lisinopril, enalapril, and most marketed ACEIs are dicarboxylate-containing, while fosinopril is the representative phosphonate-containing ACEI. All ACEIs function by inhibiting the ACE active site through chelation of the zinc ions. Lisinopril is capable of forming a hydrogen bond with E384 and Y283, establishing a salt bridge with E162 and D377, and engaging in hydrophobic interactions with K511, Q281, and Y520 of the ACE zinc-coordinating residues, as evidenced by crystal structures.^4^ According to available sources, ACEIs can be categorized into two groups: synthetic ACEIs from artificial chemical synthesis and natural product ACEIs, derived from natural sources, particularly medicinal plants.^4^

Quantitative structure–activity/property relationship (QSAR/QSPR) represents an *in silico* mathematical model used for predicting the bioactivities or properties of compounds based on their structures and physicochemical parameters.^5,6^ QSAR/QSPR is fundamentally based on two key principles: (i) the structure of a compound determines its bioactivity/property and (ii) compounds with more similar structures exhibit more similar bioactivities or properties.^6^ Particularly, structural information on compounds is represented using a range of molecular descriptors or fingerprints. These descriptors play a crucial role in determining the robustness, generalization, and predictability. QSAR/QSPR modeling can be broadly categorized into two types based on the prediction tasks: classification models and regression models.^5^

Classification models are characterized by categorical end point values, such as bioactivity potency against a target enzyme (e.g., highly active, active, intermediate, and inactive), solubility of compounds in water (e.g., highly soluble, moderately soluble, and insoluble), or liver toxicity of compounds in the human body (toxic, nontoxic). These models are established to predict the categories of bioactivity or properties. In contrast, regression models deal with numerical, continuous end point values. For example, they might predict pIC50 values of compounds targeting specific enzymes or bioavailability values of a certain category of drugs in the human body. These models aim to predict specific numerical values of the bioactivity or property.^7^

In this study, we established classification models using a variety of molecular descriptors and fingerprints. However, it is important to note that, contrary to the “similar structure, similar activity” principle, there are exceptions where structurally similar compounds exhibit significant differences in their activities against the target. This phenomenon is captured by the concept of activity cliffs (ACs), which are typically defined as pairs or groups of compounds that share close structural similarity and are active against the same pharmaceutical target but display substantial disparities in their bioactivity potency.^8^

Activity cliffs (ACs) play a crucial role in capturing chemical modifications that exert a strong influence on the biological activity. Therefore, they hold particular significance in the context of structure–activity relationship (SAR) analysis and compound optimization. However, ACs also pose a challenge to the SAR modeling process since they defy the fundamental principle that compounds with similar structures should exhibit similar bioactivity potency. Despite this challenge, ACs are highly valuable for medicinal chemists, as they provide substantial amounts of information for chemical modification and lead optimization.

In the realm of chemistry and drug discovery, QSAR/QSPR modeling techniques have become widely adopted and applied in various fields including chemistry, drug discovery, materials science, and environmental protection. As a result, OECD countries have formulated a set of principles for QSAR modeling that encompass five key rules: the requirement for a well-defined end point, the use of unambiguous algorithms, the establishment of a clearly defined applicability domain, rigorous modeling validation, and the ability to provide mechanistic interpretation.^7^ This study employed supervised machine learning approaches to construct QSAR models, in accordance with OECD principles. The outcomes of this research have the potential to contribute to the advancement of drug discovery and the optimization of ACE inhibitors (ACEIs).

## 2 Materials and Methods

**Figure 2:**
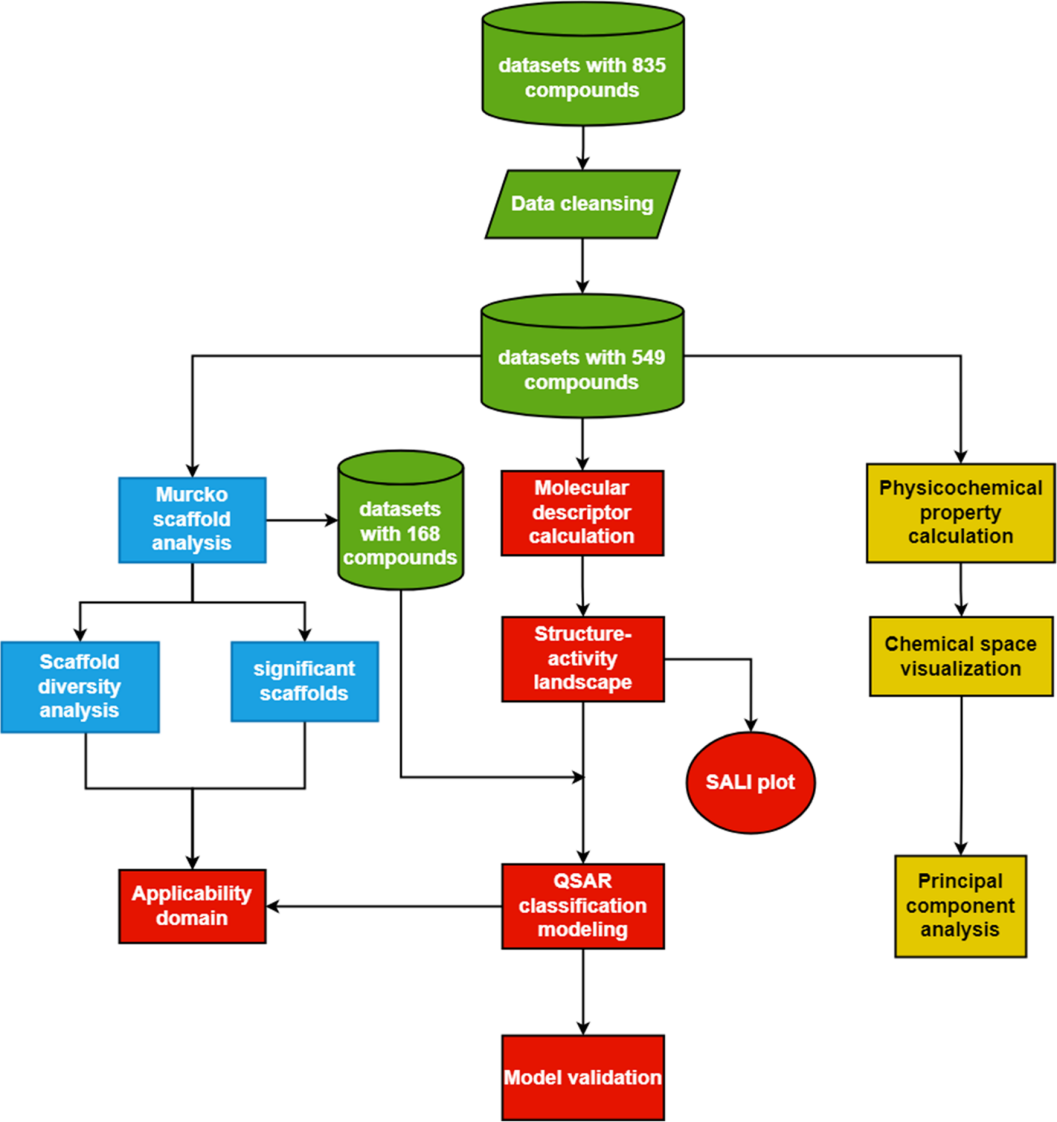
Overall workflow of this study.

The overall workflow of the study is shown in Figure 2. Particularly, different colors represent different sections: green for data collection and cleansing, blue for Murcko scaffold analysis, red for machine learning and model applicability domain determination, and yellow for exploratory data analysis.

### 2.1 Data Collection and Data Cleansing

The biological activity data of ACEIs were obtained from the ChEMBL database (ChEMBL target ID: 1808). Bioactivity data with IC_50_ values consisted of a total of 835 compounds. Subsequently, a data cleansing process was performed and this involved the removal of duplicate compounds lacking IC_50_ values or those with < or > values, as well as compounds without available SMILES annotations. This process led to a refined data set consisting of 549 compounds. To enhance the clarity and interpretability of the bioactivity values, the IC50 values were transformed into pIC50 values.^5,9^ Bioactivity transformation for classification was performed as follows: compounds with pIC_50_ > 8 were classified as active (group 1), those with pIC_50_ values between 6 and 8 were labeled as intermediate (group 2), and those with pIC_50_ values <6 were categorized as inactive (group 3). This resulted in a distribution of 148 active, 247 intermediate, and 154 inactive compounds.

To further streamline the data set and improve QSAR model performance, a subset of 168 compounds was selected based on the top 9 most prevalent Murcko scaffolds. The detailed procedure is outlined in the Murcko scaffold section. When the same bioactivity labeling criteria were applied to this subset, this yielded 24 active, 82 intermediate, and 62 inactive compounds. In the subsequent sections, all analyses and illustrations are conducted based on the data set comprising 549 compounds. In the QSAR modeling process, we adopted a dual approach. Initially, the entire data set of 549 compounds was used for modeling. Subsequently, a subset of 168 less diverse compounds was employed to refine the models.

### 2.2 Exploratory Data Analysis

A total of 8 physicochemical descriptors are calculated for exploratory data analysis. The molecular descriptors calculated are as follows: molecular weight (MW), octanol–water partition coefficient (LogP), number of hydrogen-bond acceptors (nHA), number of hydrogen-bond donors (nHD), number of rotatable bonds (nRot), topological polar surface area (TPSA), number of heteroatoms (nHET), and number of aromatic rings (Aro). Univariate statistical analysis was performed to investigate the different patterns and trends of individual molecular descriptors between 3 groups of compounds using the following descriptive statistical parameters: the minimum (Min), first quartile (Q1), median, mean, standard deviation (Std), third quartile (Q3), maximum (Max), skewness, and kurtosis. In addition, statistical differences of descriptors among active and inactive groups of compounds were evaluated using the *p*-value obtained from Student’s *t* test.

Additionally, principal component analysis (PCA) was conducted to visualize distribution patterns and compound overlaps.

### 2.3 Scaffold Analysis

#### 2.3.1 Murcko Scaffold Visualization

Murcko scaffolds and cyclic skeleton systems were extracted and compared based on pIC_50_ levels. This allowed us to identify both favorable and unfavorable scaffolds, which were subsequently used in the modeling processes. Furthermore, the frequency of the skeletons and scaffolds was also ranked. DataWarrior was employed for the generation and visualization of Murcko scaffolds.^10^ Murcko scaffold diversity is calculated as the proportion of the number of various scaffolds to the total number of compounds
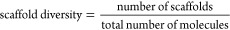


#### 2.3.2 Murcko Scaffold-Based Subset Selection

Results from the Murcko scaffold analysis played a crucial role in defining AD. Specifically, during the Murcko scaffold analysis, we selected compounds featuring the most prevalent Murcko scaffolds (with a frequency of 10 or more, totaling 9 scaffolds and 168 compounds). These selected compounds were utilized for subsequent QSAR modeling. On the other hand, compounds with less prevalent Murcko scaffolds and those exhibiting high levels of diversity and heterogeneity were excluded from this subset.

### 2.4 SALI Plot and Activity Cliffs

SALI value is a pairwise measure between the activity difference and structural difference for each pair of compounds and was calculated as eq 1, proposed by Guha and Van Drie^11^
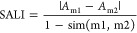
1where *A*_m1_ and *A*_m2_ are the activities of compounds 1 (abbreviated as m1) and 2 (abbreviated as m2), while sim (m1, m2) is referred to the similarity coefficient between two compounds. The SALI value increases with the possibility of the pair of compounds forming activity cliffs (ACs). In this study, this activity of compounds is represented by pIC_50_ values of compounds, while similarity is represented by the FragFP fingerprint similarity provided by DataWarrior software. DataWarrior’s default descriptor FragFp is a substructure-based binary fingerprint that consists of a dictionary of 512 predefined structure fragments. As the default molecular fingerprint in DataWarrior, it is automatically available, it does not require much space, and similarity calculations are practically instantaneous.^10^

### 2.5 QSAR Modeling

In this study, a total of 12 QSAR classification models are built for ACEIs. The modeling process consists of molecular descriptor calculation, feature selection, data balancing and splitting, machine learning modeling, applicability domain determination, and model validation. The preparation, construction, and quality control are in line with OECD principles mentioned above.

#### 2.5.1 Molecular Descriptors

In this study, Mordred descriptors were used for modeling.^12^ The open-source package contains a total of 1825 two- and three-dimensional molecular descriptors.

#### 2.5.2 Feature Selection

To improve the performance of the QSAR model and to avoid overfitting, feature selection was performed. In particular, the correlation-based filter method was employed: features with variance lower than 0.1 and features demonstrating high correlation (>0.95) were removed. In order to maintain the reproducibility of the model, the random state is set to 42.

#### 2.5.3 Data Balancing and Splitting

The working data set from the previous step of data cleansing was imbalanced between various bioactivity classes (active: 148; intermediate: 247; inactive: 154). To avoid any overfitting due to data imbalance, the data sets were then further balanced via the SMOTE oversampling technique using ScikitLearn programming, so that all three bioactivity classes have equal inputs of 247 after data balancing. In the 168 subsets of QSAR modeling (imbalanced: active: 24; intermediate: 82; inactive: 62), all three classes have equal inputs of 82 after data balancing.

Data splitting is to split the data sets into a training set and test set. By default, in the first stage of QSAR modeling (data sets with 549 input compounds), the training and test set ratio is set to 80:20. However, in the second stage (data sets with 168 input compounds), the training and test set ratio is set to 75:25 to obtain the overall better model robustness. To maintain reproducibility of the model, the random state for the data balancing and splitting is set to 42 in all procedures.

#### 2.5.4 QSAR Model Construction

Following data balancing and data splitting, a total of 12 QSAR classification models were constructed with the 6 machine learning algorithms and 2 data sets. Here, the one-vs-rest (OVR) strategy is employed for the multiclass classification. Six machine learning classification algorithms have been employed independently for model construction (Table S1 gives a complete list of algorithms). Their performances are evaluated, and the algorithm yielding the best performance will be taken.

#### 2.5.5 Applicability Domain Determination

There are 4 methods to determine the AD of a QSAR model: range-based methods, geometric methods, distance-based methods, and probability density distribution-based methods.^13,14^ In this study, there are two data sets for QSAR modeling: one is the entire data set with 549 compounds covering all of the Murcko scaffolds. Another is the subset of 168 compounds after exclusion of structurally diverse, heterogeneous compounds. The PCA bounding box method is used for AD determination of the entire 549 compounds to see whether the test set is within the boundary of the training set. For the AD determination of the 168 compounds, the scaffold-based data determine the AD: a query compound must be with the same Murcko scaffold in order to be applicable for the model.

#### 2.5.6 Model Validation

Model validation consists of both internal validation and external validation. For the internal validation, within the training set, a 10-fold cross-validation was performed to guarantee the robustness and reliability of the model. For external validation, the model is applied to test sets. Three parameters are calculated for validation: the accuracy (ac), the recall (re), and Matthew’s coefficient of correlation (mcc). Let TP, TN, FP, and FN denote true positive, true negative, false positive, and false negative, respectively. The accuracy, recall, and precision are defined as
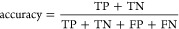

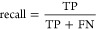

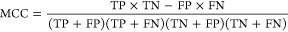


### 2.6 Reproducibility

The reproducibility of the experiment, whether *in vitro* or *in silico*, is a major concern in science and technology, as it is closely related to the extensibility of knowledge and reproducibility of outputs. As a computational study, to maintain the reproducibility of the model, all random seeds were set at 42. Data availabilities are maintained by uploading all of the data sets to the GitHub channel.

All of the above information can be accessed at https://github.com/BiochemDataWarrior/ACE-project/tree/main/Supplementals.

## 3 Results and Discussion

This section comprises a total of four subsections, each representing a distinct aspect of the study: exploratory data analysis, Murcko scaffold analysis, structure–activity relationship analysis, and SALI plot analysis.

In the exploratory data analysis, as depicted in Figure S1 and Table S1, it is evident that all eight physicochemical properties exhibit nonparametric distribution patterns. To compare the active and inactive classes, we conducted the Mann–Whitney *U* test to assess the statistical significance. Following the *U* test, it was observed that all eight properties, except for LogP and Aro, demonstrated statistical significance. Specifically, compounds from the active class typically exhibited higher values for MW (molecular weight), nHA (number of heavy atoms), nRot (number of rotatable bonds), TPSA (topological polar surface area), and nHET (number of heteroatoms) when compared to those of the inactive class.

**Figure 3:**
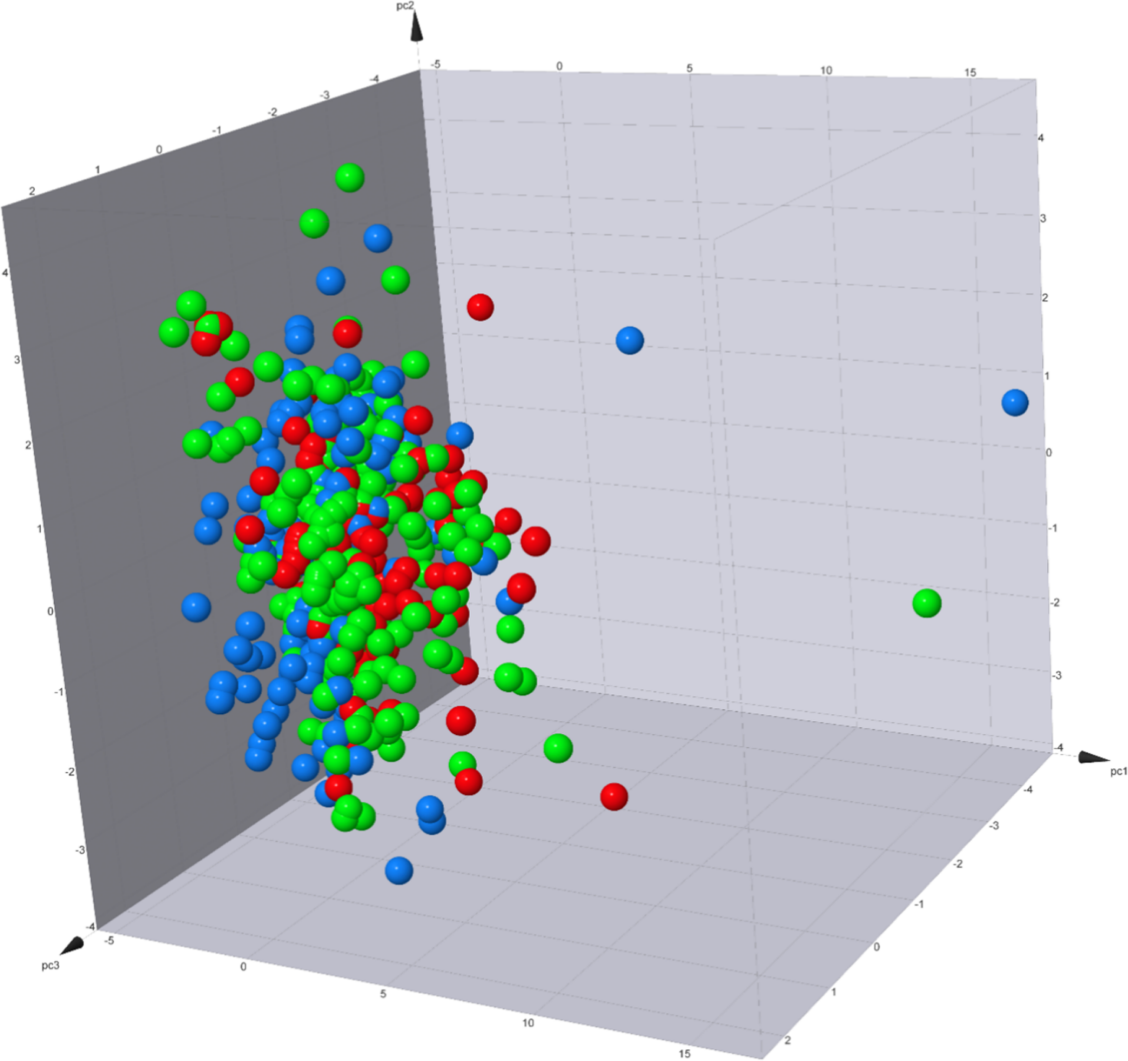
Principal component analysis of 8 physicochemical properties with ACE inhibitors represented as spherical markers. Bioactivity classes are color-coded: red for active, green for intermediate, and blue for inactive. The *X*-axis corresponds to PC1, the *Y*-axis corresponds to PC2, and the *Z*-axis corresponds to PC3.

The PCA plot, which incorporates the eight physicochemical properties, revealed significant overlap among the three bioactivity classes, as depicted in Figure 3. Furthermore, the eigenvalues associated with these eight properties shed light on their contributions to the principal components (PCs). PC1 was primarily influenced by nHET (0.433) and nHA (0.421), followed closely by TPSA (0.396), MW (0.395), nHD (0.373), and nRot (0.368). PC2 exhibited the highest loadings for LogP (0.665), Aro (0.573), and MW (0.343), while nHA and nHD were the most significant negative contributors. The third principal component (PC3) had the highest loading from Aro (0.683) and nHD (0.468), with nRot (−0.351) being the most significant negative contributor. This information is visually represented in Figure S2 and Table 1.

**Table 1:** Eigenvalues of the 8 Physicochemical Properties

property	PC1	PC2	PC3
MW	0.395	0.343	–0.138
LogP	–0.145	0.665	–0.314
nHA	0.421	–0.131	0.012
nHD	0.373	–0.208	0.468
TPSA	0.396	–0.089	–0.234
nRot	0.368	0.192	–0.351
nHET	0.433	–0.078	–0.137
Aro	0.162	0.573	0.683
cumulated variance (%)	59.933	81.565	89.25

### 3.1 Exploratory Data Analysis

Physicochemical property exploration is the initial analysis of the compounds at the general level. To get more specific information, the compounds should be investigated furthermore, and then scaffold analysis follows.

### 3.2 Scaffold Analysis

**Figure 4:**
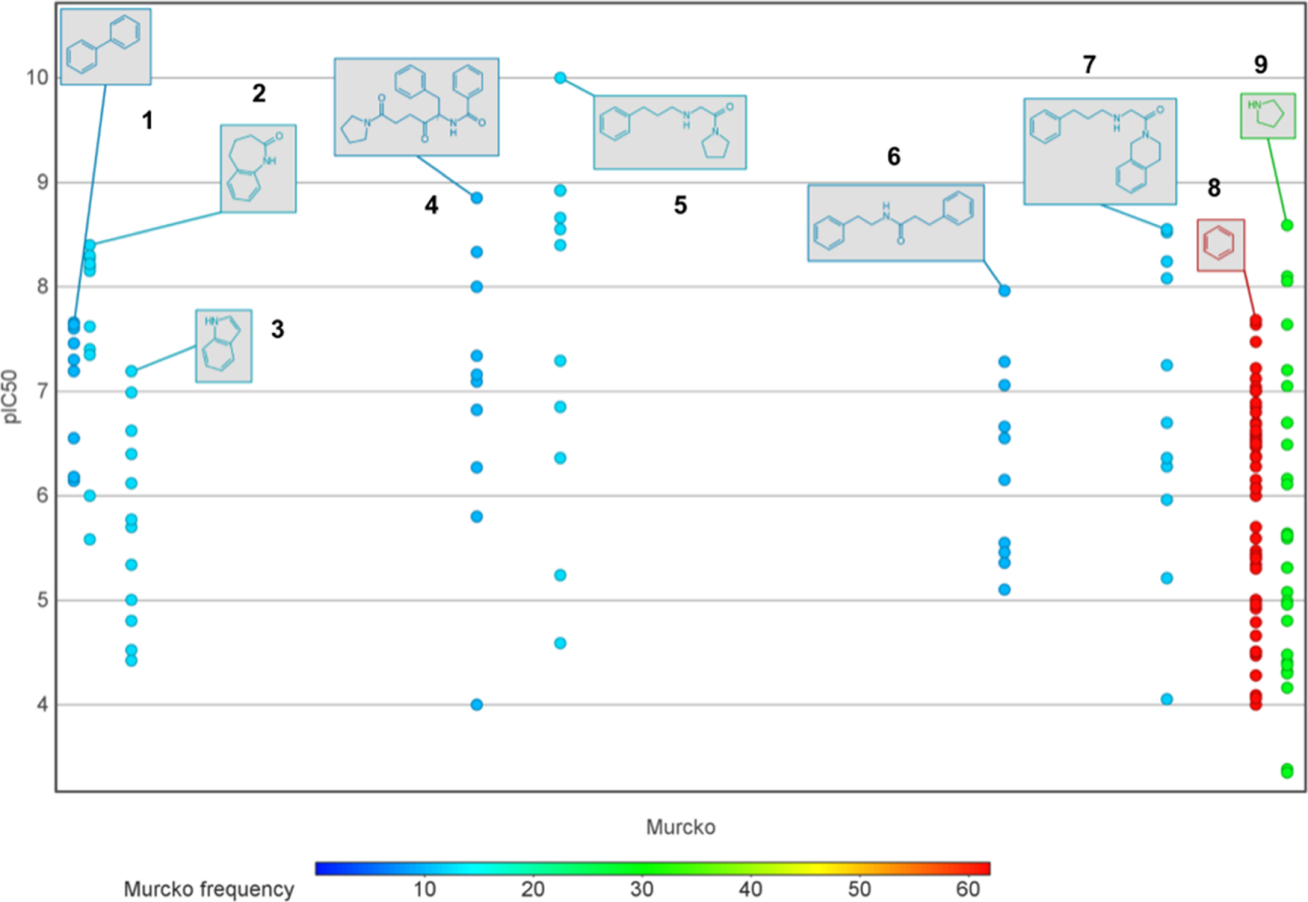
Scaffold plot from DataWarrior. The Murcko scaffold underlying each compound was extracted, encoded by SMILES notations, and calculated for their frequencies. In addition, the Murcko scaffolds were correlated with pIC_50_ levels. In this figure, the colors represent various Murcko scaffold frequencies.

Scaffold analysis aims to visualize the Murcko scaffolds and analyze their diversities. In addition, scaffolds combined with bioactivities can provide straightforward insights into the scaffold–activity relationship landscapes. Table 2 shows the scaffold diversity among different subsets of ACE inhibitor compounds. Among the 549 compounds, there are 239 Murcko scaffolds and 169 Murcko skeletons (cyclic skeleton system, which is the generalized form of the Murcko scaffold). Among the 239 Murcko scaffolds, there are only 9 of them with frequency ≥10 as shown in Figure 4. Seen from the scatters, scaffolds 2, 4, 5, 7, and 9 have compounds with pIC_50_ > 8, while compounds with scaffolds 1, 3, 6, and 8 all have pIC_50_ < 8. It is noteworthy to mention that captopril is the representative drug with scaffold 9, benazepril with scaffold 2, enalapril and lisinopril with scaffold 5, and moexipril and quinapril with scaffold 7.

**Table 2:** Murcko Scaffold Diversity Analysis^a^

	number of compounds (*N*)	Murcko scaffold (Ns)	cyclic skeletons (Ncsk)	Ns/N	Ncsk/N	Ncsk/Ns
complete	549	239	169	0.435	0.308	0.707
active	148	102	73	0.689	0.493	0.716
intermediate	247	129	99	0.522	0.401	0.767
inactive	154	67	57	0.435	0.37	0.851

a Scaffold diversity is calculated as the proportion of the number of scaffolds to the total number of compounds. In this table, *N* means the number of total compounds, Ns for the number of Murcko scaffolds, and Ncsk for the number of cyclic skeletons.

Generally, compounds in the active class demonstrate scaffold diversity that is higher than that of compounds in the inactive class. Therefore, there is potential to find more novel compounds based on the active groups for future ACE inhibitors. More derivatives based on these scaffolds should be synthesized and explored for bioactivities against ACE.

Scaffold analysis has revealed the scaffold–activity relationship landscape. This is a preliminary investigation into the structure–activity relationship of ACEIs. To dive deeper and more comprehensively, machine learning-based structure–activity relationships of ACEIs are executed.

### 3.3 QSAR Modeling

A total of 6 + 6 (former 6 for the data sets with 549 compounds and latter 6 for data sets with 168 compounds covering the 9 representative Murcko scaffolds) QSAR models have been established on Mordred descriptors along with 6 well-defined algorithms (Extra Trees, Random Forest, LightGBM, Extreme gradient boost, Multilayer perceptron (MLP), Gaussian Process). The whole modeling processes are executed on the Jupyter notebook with Python programming language. Table 3 shows the summary of best model performances with two sets of data. It is concluded that for the entire data set with 549 compounds, Random Forest (RF) algorithm provides the best model performance, with accuracy 0.981 in the training set, 0.77 in the 10-fold cross-validation set, and 0.745 in the testing set. For the 168 subset compounds, multilayer perceptron (MLP) algorithm is the best with accuracy 0.973 in the training set, 0.815 in the 10-fold cross-validation set, and 0.806 in the testing set. All of the model performances using other algorithms are listed in Table S3.

**Table 3:** QSAR Model Performance Metric Using Mordred Descriptors^a^

	accuracy			recall			MCC		
	train	CV	test	train	CV	test	train	CV	test
all 549 compounds, RF	0.981	0.77	0.745	0.981	0.771	0.745	0.972	0.658	0.617
168 subset compounds, MLP	0.973	0.832	0.823	0.973	0.833	0.818	0.959	0.762	0.737

a Data sets with 549 compounds without the exclusion of Murcko scaffolds.

**Figure 5:**
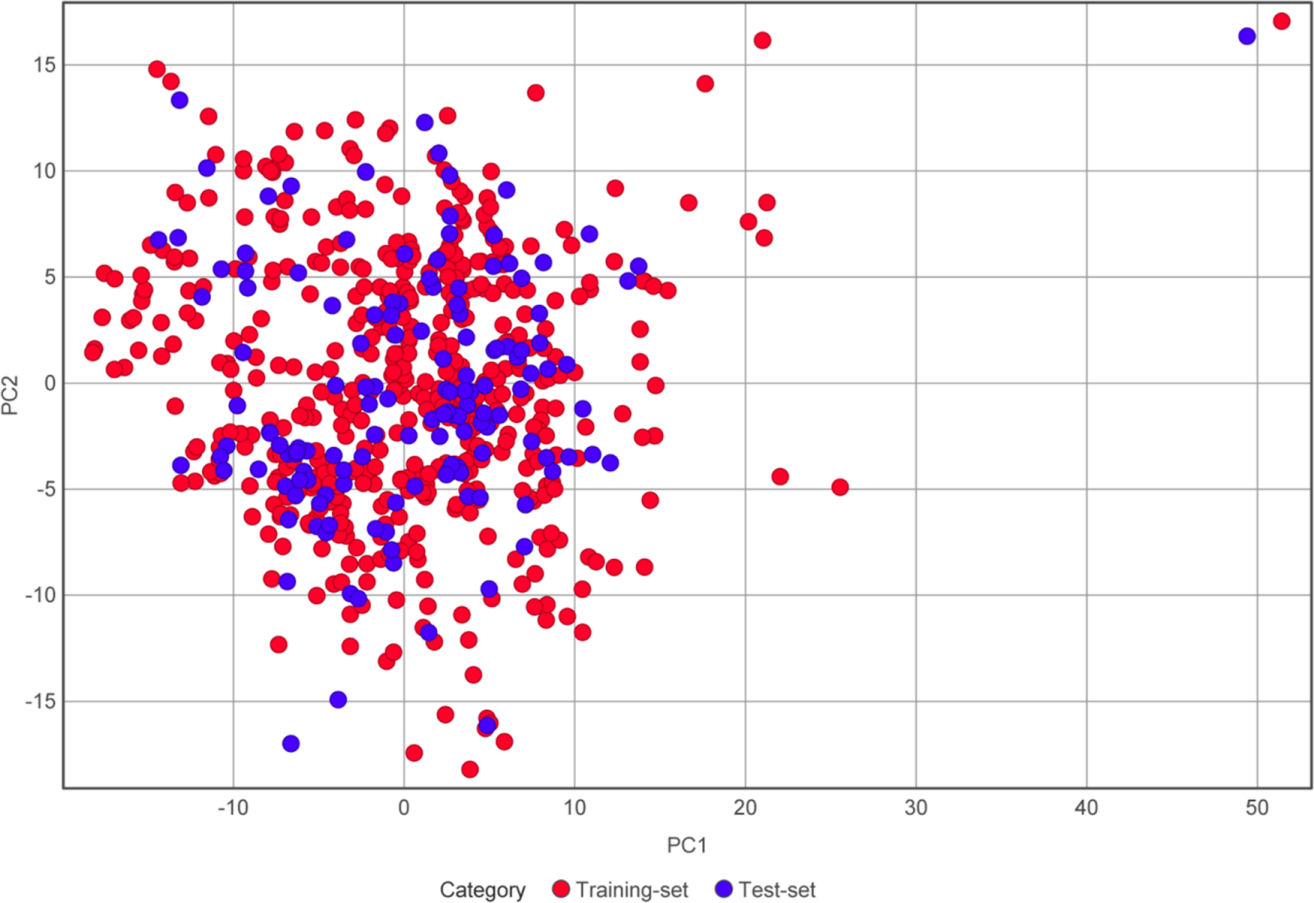
Applicability domain visualization of the QSAR model with the PCA bounding box.

The applicability domain of the model is visualized by the PCA bounding box. As seen from Figure 5, test set compounds are mostly distributed within the bounding area of training set compounds. Future application of the model can be predetermined by scaffold analysis in combination with the PCA bounding box. To be specific, for a query compound, the Murcko scaffold needs to be extracted to see whether it matches the scaffold present in this model. If there is no match, then the query compound should not be evaluated with this model. If there is a match, then the compound should be checked for its coordinates in the PCA bounding box.

**Figure 6:**
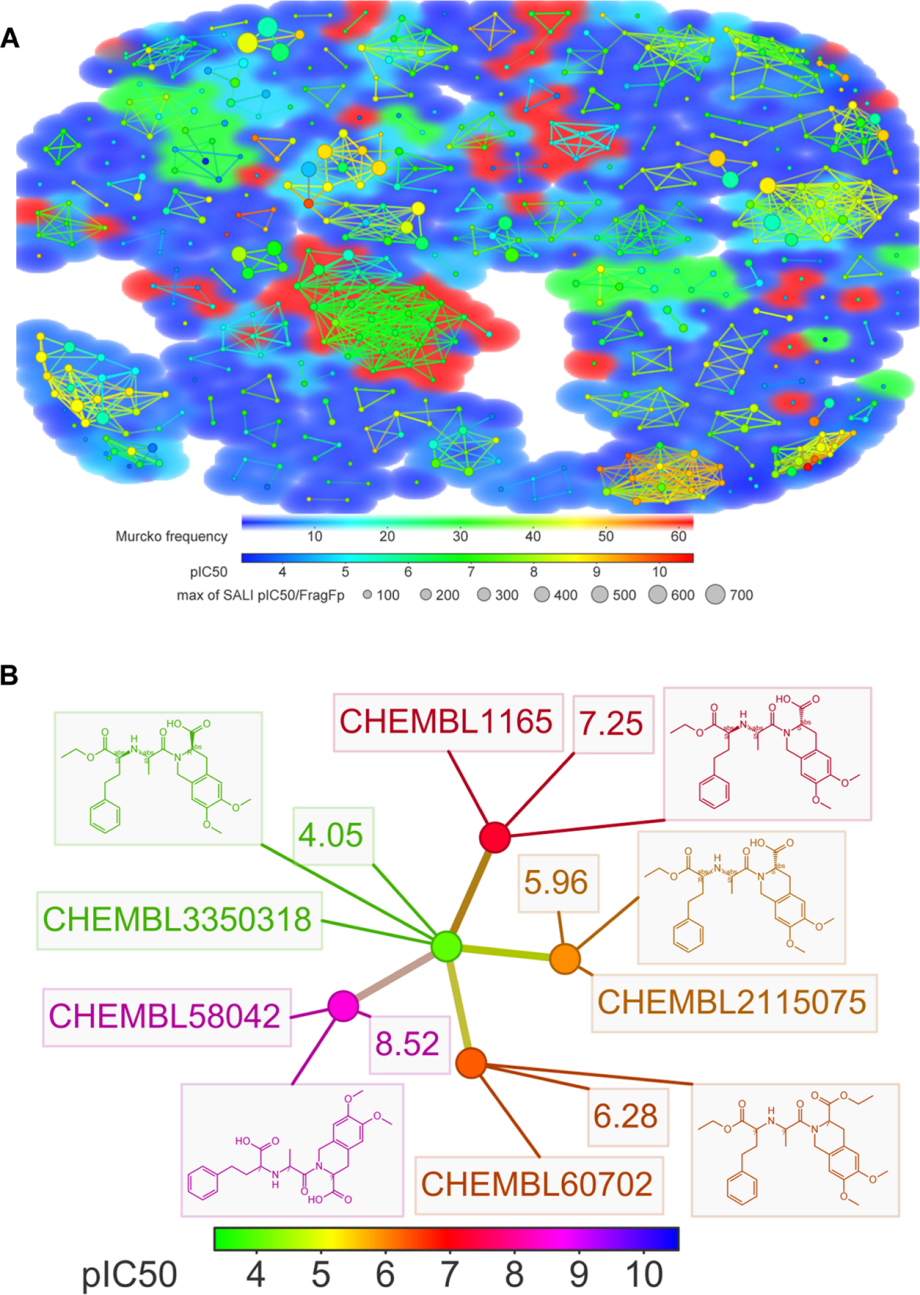
SALI plot for all ACE inhibitors explored in the study (A) and the neighbor network of a representative compound (B). (A) The color of the nodes represents the pIC50 values of each compound, the background color corresponds to the frequency of the Murcko scaffold for that compound, and the sizes of the nodes reflect the SALI value for each pair of compounds.

The QSAR models in this section can be used for bioactivity prediction and drug optimizations. To illustrate the structure–activity relationships, as well as identify ACs, a SALI plot facilitated by DataWarrior software is generated. In the plot, the complicated network of the ACEIs can be clearly visualized and ACs can be easily identified as depicted in Figure 6.

### 3.4 SALI Plot and Activity Cliffs

Figure 6A shows the SALI plot for all of the ACE inhibitors of this study, generated by DataWarrior software. Each marker represents an individual compound from the data sets. The color for the nodes represents the pIC_50_ values, the marker background colors represent the Murcko frequency of the individual compound, and the size of the marker represents the SALI value for its corresponding neighbor compounds. The whole SALI plot is the complete visualization of the SAR and the connection networks between the compounds. From the SALI plot, we can clearly recognize clusters of structurally similar compounds, identify compounds of various pIC_50_ levels (blue–green–red), locate ACs (large markers), and distinguish the cluster of compounds with different Murcko scaffolds. The neighbor network of the representative compounds in Figure 6B is the visualization of the relationships and bioactivity differences. Illustrated in Figure 6B is the structure–activity relationship network holding AC. All of the compounds in this network belong to the Murcko scaffold 7 series. The center of the network is a compound (ChEMBL 3350318) with very weak bioactivity (pIC_50_ = 4.05). However, a minor chemical modification on the stereochemistry of the functional group can enlarge bioactivity potency levels significantly by up to 10^4^ magnitudes. The compound (ChEMBL 3350318) forms four pairs of ACs with ChEMBL 2115075, 60702, 1165, and 58042. In Figure 6A, the proportion of bigger-sized markers represents a very minority of all of the networks. Therefore, a marker compound exemplified by ChEMBL 3350318 can be highly rare and informative as an AC generator. Markers that form multiple ACs can be highly valuable and informative for lead optimizations.

**Figure 7:**
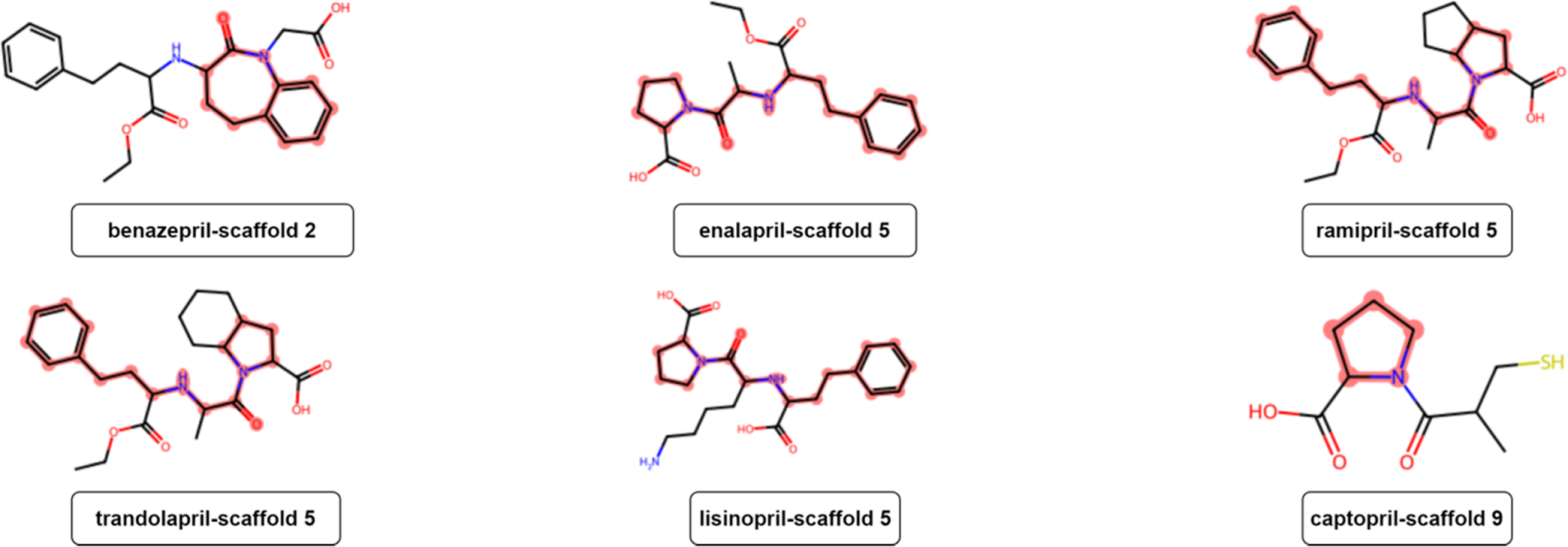
Murcko scaffold highlights structures of representative ACEIs. The red color indicates the Murcko scaffolds as represented in the ACEIs.

According to the Murcko scaffold analysis conducted in this study, captopril belongs to scaffold 9, benazepril: scaffold 2; enalapril, lisinopril, ramipril, and trandolapril: scaffold 5 as shown in Figure 7. The 9 most prevalent 9 Murcko scaffolds demonstrated in Figure 4 cannot cover all of the marketed ACEIs; meanwhile, when correlated with pIC_50_ levels, we can identify favorable and unfavorable scaffolds: the higher proportion of compounds incorporating the given scaffold have higher overall pIC_50_ levels, the more favorable it would be. Therefore, scaffolds 2, 4, 5, 7, and 9 are more favorable than scaffolds 1, 3, 6, and 8. In particular, ACEIs with scaffolds 1, 3, 6, and 8 have no candidate that belongs to the active bioactivity class (pIC_50_ ≥ 8). On the contrary, among the 9 scaffolds, scaffold 5 is more favorable than the other favorable scaffolds (2, 4, 7, and 9) because this scaffold has the candidate ACEI with the highest pIC_50_ level (pIC_50_ = 10).

Until now, there have been a number of studies on the structure–activity relationships of ACE inhibitors.^15−18^ Most of them are focused on bioactive peptides instead of miscellaneous small compounds. This study is focused on small compounds that target ACE.

This study is a computational study focusing on the chemical space and structure–activity relationships of ACE inhibitors. Using comprehensive data from the ChEMBL database, the study has identified the physicochemical properties, Murcko scaffolds, diversity, and distribution of ACE inhibitors. In addition, there are a total of 12 QSAR models for ACE inhibitors using Mordred descriptors. Using the SALI plot to visualize the SAR landscape, the neighbor network of the compounds is clarified. The findings in this study can facilitate further drug discovery and optimization of ACE inhibitors to manage hypertension and chronic heart failure.

Based on the current knowledge gap and challenges in drug discovery, clinical applications, to better optimize ACEIs, especially to identify selective C-domain or N-domain inhibitory ACEIs, we have three future recommendations: first, using computational drug discovery, especially machine learning approaches, to facilitate the currently available ACEI chemical modification; second, more and better crystal structures of human ACE and binding complexes with various ACEIs should be revolved. As one of the starting points of computational drug discovery, high-quality crystal structures of the target protein and binding complexes are highly valuable for the investigation of ligand–protein interaction profiles and virtual screening. Finally, there are lots of natural products, from plants, animals, microorganisms, and marine environments, that prove to be potential hits that target ACE. Phytochemicals such as luteolin, quercetin-3-*O*-*b*-d-galactopyranoside, quercetin-3-*O*-a-l-arabinofuranoside, and myricetin-3-*O*-(600-*O*-galloyl)-*b*-d-glucopyranoside from *L. michelsonii* all have IC_50_ against ACE for less than 20 uM.^19^ In addition, marine peptides such as MLVFAV from *Skipjack*,^20^ GASSGMPG from Pacific cod,^21^ and VPAAPPK and NGTWFEPP from *Thermolysin*(22) all demonstrate IC_50_ against ACE for less than 10 uM. The abundance of natural products and their structural diversities provide them with promising pharmacodynamic and pharmacokinetic properties. Natural products should be alternative considerations for further drug discovery.

A limitation of this study is that it is a computational study without an experimental application. This can be performed in future studies on cell culture and animal models. In addition, the source of data is the retrospective documentation of previous experiments. Due to the retrospective nature, the ongoing experimental data that may be of interest are not included.

## 4 Conclusions

The discovery of captopril, the first ACE inhibitor, has paved the way for the development of numerous ACE inhibitors used in the treatment of conditions such as hypertension, chronic heart failure, and kidney diseases. This computational study is focused on enhancing ACE inhibitor discovery and optimization through exploratory data analysis, which allowed the visualization of the physicochemical properties of compounds. As such, this allowed the exploration of distributions, patterns, and statistical significance among the bioactivity groups. Murcko scaffold analysis allowed the identification of 9 key scaffolds with frequencies exceeding 10, highlighting greater scaffold diversity in active ACE inhibitors. Scaffold diversity analysis revealed that active-class ACE inhibitors exhibited greater scaffold diversity than their intermediate and inactive counterparts, suggesting a substantial opportunity for lead optimization within the active-class ACE inhibitors. Moreover, our correlation analysis between Murcko scaffold types and their bioactivities led to the conclusion that certain scaffolds (1, 3, 6, and 8) are less favorable when compared with alternative scaffolds (2, 3, 5, 7, and 9). In our subsequent QSAR modeling, we harnessed a data set comprising the 9 representative Murcko scaffold-matched compounds, totaling 168, for further analysis. Through this approach, we achieved robust model performance utilizing Mordred descriptors and the Extremely Gradient Boost algorithm. Our models exhibited robust accuracy scores of 0.973, 0.849, and 0.823, with corresponding MCC values of 0.959, 0.786, and 0.742 in the training set, 10-fold cross-validation set, and testing set, respectively. Furthermore, additional insight into the structure–activity relationship was gained through SALI plots, revealing clusters of compounds forming activity cliffs. The outcomes of the Murcko scaffold analysis offer valuable guidance for optimizing medicinal chemistry, while the establishment of QSAR models enhances our ability to predict ACE inhibitor bioactivity classes. The SALI plot also contributes vital insights into activity cliff information, thus serving as a guiding tool in the realms of drug discovery and optimization. However, it is essential to acknowledge that this study is currently limited to a computational approach without experimental validation. In the future, the knowledge gained from the Murcko scaffold analysis, SALI plots, and QSAR models can be leveraged in cell and animal experiments to design more effective ACE inhibitors.

## Data Availability

The data sets and source codes underlying this article are available at GitHub: https://github.com/BiochemDataWarrior/ACE-project

## Supporting Information Available

Comprehensive overview of the machine learning algorithms and exploratory data analysis procedures used in this study: machine learning algorithms for modeling (Table S1); exploratory data analysis of 8 physicochemical properties and comparison between bioactivity classes, *p*-value denotes the Mann–Whitney *U* test result (Table S2); QSAR model performance metric using Mordred descriptors (Table S3); comparison of eight physicochemical properties between bioactivity classes (Figure S1); and eigenvalues of 8 properties (Figure S2) (PDF)

## References

[ref1] Polakovičová M.; Jampílek J. Advances in Structural Biology of ACE and Development of Domain Selective ACE-inhibitors. Med. Chem. 2019, 15 (6), 574–587. 10.2174/1573406415666190514081132. 31084594

[ref2] Song C. C.; Qiao B. W.; Zhang Q.; Wang C. X.; Fu Y. H.; Zhu B. W. Study on the domain selective inhibition of angiotensin-converting enzyme (ACE) by food-derived tyrosine-containing dipeptides. J. Food Biochem. 2021, 45 (7), e13779 10.1111/jfbc.13779. 34060658

[ref3] Helmer A.; Slater N.; Smithgall S. A Review of ACE Inhibitors and ARBs in Black Patients With Hypertension. Ann. Pharmacother. 2018, 52 (11), 1143–1151. 10.1177/1060028018779082. 29808707

[ref4] Zheng W.; Tian E.; Liu Z.; Zhou C.; Yang P.; Tian K.; Liao W.; Li J.; Ren C. Small molecule angiotensin converting enzyme inhibitors: A medicinal chemistry perspective. Front. Pharmacol. 2022, 13, 968104 10.3389/fphar.2022.968104. 36386190 PMC9664202

[ref5] Nantasenamat C. Best Practices for Constructing Reproducible QSAR Models. In Ecotoxicological QSARs; Roy K., Ed.; Springer US, 2020; pp 55–75.

[ref6] Yu T.; Nantasenamat C.; Kachenton S.; Anuwongcharoen N.; Piacham T. Cheminformatic Analysis and Machine Learning Modeling to Investigate Androgen Receptor Antagonists to Combat Prostate Cancer. ACS Omega 2023, 8 (7), 6729–6742. 10.1021/acsomega.2c07346. 36844574 PMC9948163

[ref7] Fjodorova N.; Novich M.; Vrachko M.; Smirnov V.; Kharchevnikova N.; Zholdakova Z.; Novikov S.; Skvortsova N.; Filimonov D.; Poroikov V. Directions in QSAR modeling for regulatory uses in OECD member countries, EU and in Russia. J. Environ. Sci. Health, Part C: Environ. Carcinog. Ecotoxicol. Rev. 2008, 26 (2), 201–236. 10.1080/10590500802135578. 18569330

[ref8] Stumpfe D.; Hu H.; Bajorath J. Evolving Concept of Activity Cliffs. ACS Omega 2019, 4 (11), 14360–14368. 10.1021/acsomega.9b02221. 31528788 PMC6740043

[ref9] Schaduangrat N.; Lampa S.; Simeon S.; Gleeson M. P.; Spjuth O.; Nantasenamat C. Towards reproducible computational drug discovery. J. Cheminf. 2020, 12 (1), 9 10.1186/s13321-020-0408-x. PMC6988305 33430992

[ref10] Sander T.; Freyss J.; von Korff M.; Rufener C. DataWarrior: an open-source program for chemistry aware data visualization and analysis. J. Chem. Inf. Model. 2015, 55 (2), 460–473. 10.1021/ci500588j. 25558886

[ref11] Guha R.; Van Drie J. H. Structure--activity landscape index: identifying and quantifying activity cliffs. J. Chem. Inf. Model. 2008, 48 (3), 646–658. 10.1021/ci7004093. 18303878

[ref12] Moriwaki H.; Tian Y. S.; Kawashita N.; Takagi T. Mordred: a molecular descriptor calculator. J. Cheminf. 2018, 10 (1), 4 10.1186/s13321-018-0258-y. PMC5801138 29411163

[ref13] Sahigara F.; Mansouri K.; Ballabio D.; Mauri A.; Consonni V.; Todeschini R. Comparison of different approaches to define the applicability domain of QSAR models. Molecules 2012, 17 (5), 4791–4810. 10.3390/molecules17054791. 22534664 PMC6268288

[ref14] Roy K.; Ambure P.; Kar S. How Precise Are Our Quantitative Structure-Activity Relationship Derived Predictions for New Query Chemicals?. ACS Omega 2018, 3 (9), 11392–11406. 10.1021/acsomega.8b01647. 31459245 PMC6645132

[ref15] Deng B.; Ni X.; Zhai Z.; Tang T.; Tan C.; Yan Y.; Deng J.; Yin Y. New Quantitative Structure-Activity Relationship Model for Angiotensin-Converting Enzyme Inhibitory Dipeptides Based on Integrated Descriptors. J. Agric. Food Chem. 2017, 65 (44), 9774–9781. 10.1021/acs.jafc.7b03367. 28984136

[ref16] Sun H.; Chang Q.; Liu L.; Chai K.; Lin G.; Huo Q.; Zhao Z.; Zhao Z. High-Throughput and Rapid Screening of Novel ACE Inhibitory Peptides from Sericin Source and Inhibition Mechanism by Using in Silico and in Vitro Prescriptions. J. Agric. Food Chem. 2017, 65 (46), 10020–10028. 10.1021/acs.jafc.7b04043. 29086555

[ref17] Tripaldi P.; Pérez-González A.; Rojas C.; Radax J.; Ballabio D.; Todeschini R. Classification-based QSAR Models for the Prediction of the Bioactivity of ACE-inhibitor Peptides. Protein Pept. Lett. 2018, 25 (11), 1015–1023. 10.2174/0929866525666181114145658. 30430931

[ref18] Wang F.; Zhou B. Insight into structural requirements of ACE inhibitory dipeptides: QSAR and molecular docking studies. Mol. Diversity 2020, 24 (4), 957–969. 10.1007/s11030-019-10005-0. 31655961

[ref19] Jenis J.; Kim J. Y.; Uddin Z.; Song Y. H.; Lee H. H.; Park K. H. Phytochemical profile and angiotensin I converting enzyme (ACE) inhibitory activity of Limonium michelsonii Lincz. J. Nat. Med. 2017, 71 (4), 650–658. 10.1007/s11418-017-1095-4. 28550653

[ref20] Intarasirisawat R.; Benjakul S.; Wu J.; Visessanguan W. Isolation of antioxidative and ACE inhibitory peptides from protein hydrolysate of skipjack (Katsuwana pelamis) roe. J. Funct. Foods 2013, 5 (4), 1854–1862. 10.1016/j.jff.2013.09.006.

[ref21] Daskaya-Dikmen C.; Yucetepe A.; Karbancioglu-Guler F.; Daskaya H.; Ozcelik B. Angiotensin-I-Converting Enzyme (ACE)-Inhibitory Peptides from Plants. Nutrients 2017, 9 (4), 316 10.3390/nu9040316. 28333109 PMC5409655

[ref22] Ghassem M.; Arihara K.; Babji A. S.; Said M.; Ibrahim S. Purification and identification of ACE inhibitory peptides from Haruan (Channa striatus) myofibrillar protein hydrolysate using HPLC-ESI-TOF MS/MS. Food Chem. 2011, 129 (4), 1770–1777. 10.1016/j.foodchem.2011.06.051.

