# Supplementary material for: Machine Learning Approaches to Investigate the Structure–Activity Relationship of Angiotensin-Converting Enzyme Inhibitors
Source: ACS Omega. 2023 Nov 8;8(46):43500–10. doi: 10.1021/acsomega.3c03225 (PMC10666249; doi:10.1021/acsomega.3c03225)
Supplement: Supplementary file 1 — ao3c03225_si_001.pdf [file ao3c03225_si_001.pdf]

MACHINE LEARNING APPROACHES TO INVESTIGATE THE STRUCTURE-ACTIVITY RELATIONSHIP OF ANGIOTENSIN-CONVERTING ENZYME INHIBITORS

Tianshi Yu[1], Chanin Nantasenamat[2], Nuttapat Anuwongcharoen[1], Theeraphon Piacham[3*]

[1] Center of Data Mining and Biomedical informatics, Faculty of Medical Technology, Mahidol University, Bangkok 10700, Thailand

[2] Streamlit Open Source, Snowflake Inc., San Mateo, California 94402, United States

[3] Department of Clinical Microbiology and Applied Technology, Faculty of Medical Technology, Mahidol University, Bangkok 10700, Thailand

Corresponding Author:

Theeraphon Piacham*

Department of Clinical Microbiology and Applied Technology, Faculty of Medical Technology, Mahidol University, Bangkok, Thailand

Email address: theeraphon.pia@mahidol.ac.th

Table S1. Machine learning algorithms for modeling

Algorithm	Abbr	Type	Description
**Extra trees**	ET	Ensemble learning	*max_features = 5*<br>*n_estimators = 1000*<br>*random state = 42*
**Random forest**	RF	Ensemble learning	*max_features = 5*

			n_estimators = 1000
			random state = 42
			criterion = gini
**LightGBM**	LGBM	Ensemble learning	*n_estimators = 1000*
			random state = 42
**Extreme gradient boost**	XGB	Ensemble learning	*n_estimators = 1000*
			random state = 42
**Multilayer perceptron**	MLP	Artificial neural network	*Activation = relu*
			Solver = adam
			Learning rate = constant
			hidden_layer_sizes = 512
			random state = 42
**Gaussian process**	GP	Non-parametric	*max_iter_predict=500*
			multi-class=one_vs_rest
			random state = 42

Table S2. Exploratory data analysis of 8 physicochemical properties and comparison between bioactivity classes. The p-value denotes the Mann-Whitney U test result.

	**MW**		**LogP**		**nHA**		**nHD**	
	**active**	**inactive**	**active**	**inactive**	**active**	**inactive**	**active**	**inactive**

***p*-value**	4.01332667e-11		0.15113827		1.37885476e-09		0.00019855	
**min**	217.29	162.21	-5.67	-7.10	4	1	1	0
**Q1**	368.24	259.58	-0.86	-1.09	7	4	2	2
**median**	413.46	322.45	-0.13	0.65	7	6	3	2
**mean**	422.54	348.45	0.38	0.67	7.53	6.14	2.83	2.37
**std**	79.47	124.63	1.96	2.47	2.05	2.57	1.15	1.28
**Q3**	468.91	429.56	1.59	2.72	9	7	3	3
**max**	703.80	1174.44	5.35	5.32	16	25	8	8
**skew**	0.34	2.30	0.24	-0.36	0.72	2.81	1.04	0.92
**kurtosis**	0.99	12.23	0.15	-0.24	2.20	17.79	3.17	2.02

	**TPSA**		**nRot**		**nHET**		**Aro**	
	**active**	**inactive**	**active**	**inactive**	**active**	**inactive**	**active**	**inactive**
***p*-value**	7.03534658e-09		8.51495926e-05		1.04832227e-12		0.02483612	
**min**	26.36	38.77	2	0	5	2	0	0
**Q1**	106.94	97.33	7	5	7	5	1	0

**median**	127.25	108.21	8	7	8	6	1	1
**mean**	130.39	115.80	8.92	7.34	8.26	6.87	1.36	1.14
**std**	26.36	33.93	3.46	4.07	1.86	2.47	0.79	0.90
**Q3**	137.02	126.82	11	10	9	8	2	2
**max**	239.98	351.81	17	36	16	25	4	4
**skew**	1.62	2.95	0.30	2.38	1.29	3.13	0.66	0.38
**kurtosis**	3.88	17.33	-0.47	14.92	3.01	19.02	1.29	-0.37

Table S3. QSAR model performance metric using Mordred descriptors.

	**Accuracy**			**Recall**			**MCC**		
	**Train**	**CV**	**Test**	**Train**	**CV**	**Test**	**Train**	**CV**	**Test**
**ET**	0.973	0.827	0.79	0.972	0.829	0.794	0.96	0.756	0.693
**RF**	0.973	0.822	0.774	0.972	0.824	0.78	0.959	0.752	0.673
**LGBM**	0.973	0.838	0.774	0.972	0.839	0.78	0.96	0.767	0.664
**XGB**	0.973	0.849	0.823	0.973	0.852	0.822	0.959	0.786	0.742
**MLP**	0.973	0.827	0.806	0.973	0.827	0.808	0.959	0.752	0.712
**GP**	0.973	0.337	0.435	0.974	0.353	0.386	0.96	0.066	0.188

Figure S1. Comparison of eight physicochemical properties between bioactivity classes.

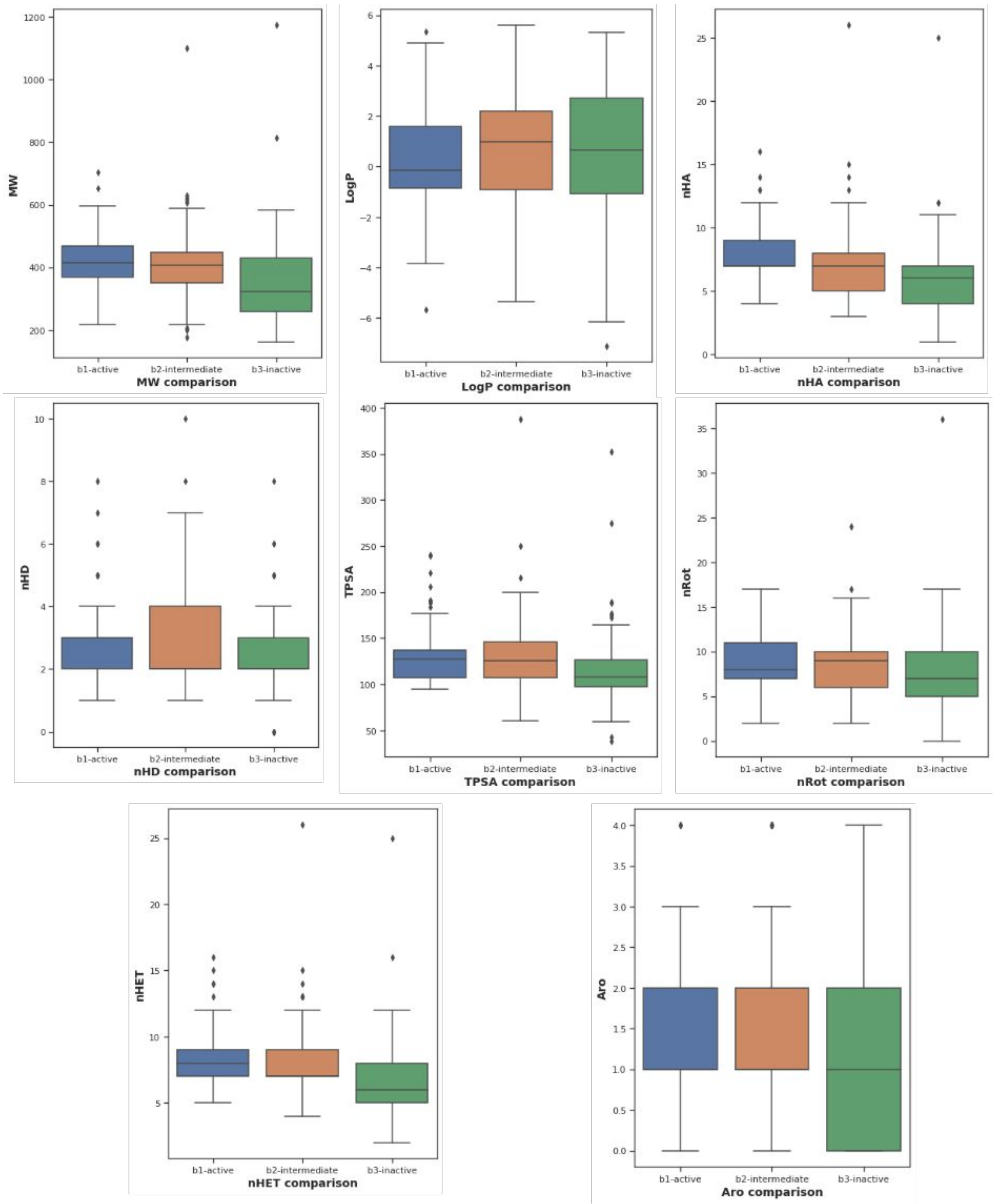

Figure S2. Eigenvalues of eight physicochemical properties

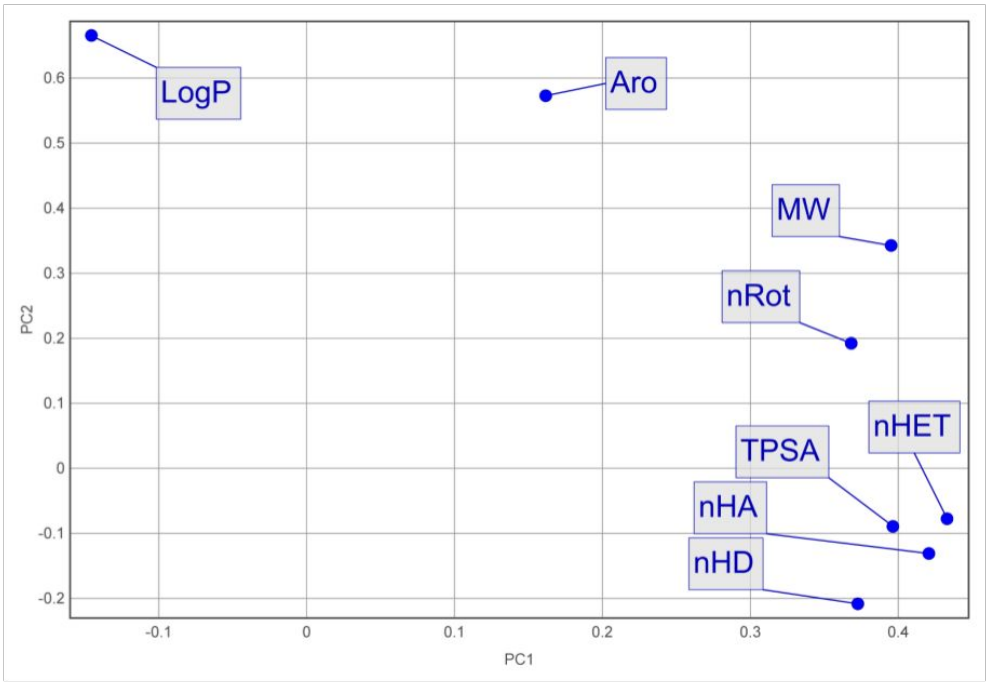
LogP
Aro
MW
nRot
nHET
TPSA
nHA
nHD
PC1
PC2
0.6
0.5
0.4
0.3
0.2
0.1
0
-0.1
-0.2
-0.1
0
0.1
0.2
0.3
0.4
